# Adolescents’ narratives of coping with unintended pregnancy in Nairobi’s informal settlements

**DOI:** 10.1371/journal.pone.0240797

**Published:** 2020-10-29

**Authors:** Joyce N. Mumah, Stephen Mulupi, Yohannes D. Wado, Boniface A. Ushie, Deladem Nai, Caroline W. Kabiru, Chimaraoke O. Izugbara

**Affiliations:** 1 African Population and Health Research Center, Nairobi, Kenya; 2 Population Council, Accra, Ghana; 3 School of Public Health, University of Witwatersrand, Johannesburg, South Africa; 4 International Center for Research on Women (ICRW), Washington, DC, United States of America; Kwame Nkrumah University of Science and Technology, GHANA

## Abstract

**Aim:**

This study explored adolescent experiences and coping strategies for unintended pregnancy in two informal settlements—Viwandani and Korogocho—in Nairobi, Kenya.

**Methods:**

Forty-nine in-depth-interviews and eight focus group discussions were conducted with male and female adolescents aged 15–19 years from households in two informal settlements. Participants were purposively selected to include adolescents of varying socio-demographic characteristics, including the married and unmarried, and adolescents who had never/ever been pregnant. Data were transcribed, translated verbatim and analyzed thematically.

**Results:**

Adolescents attributed unintended pregnancy to poverty, sexual violence and inconsistent contraceptive use. Lack of parental support and guidance, as well as household conflicts also exposed girls to early sexual debut and risky sexual behavior. Decisions about pregnancy management centered on carrying the pregnancy to term or terminating it. Deciding to terminate a pregnancy was not always straightforward and was motivated by concerns about stigma or shame, and school disruption. Participants reiterated that carrying an unintended pregnancy to term disrupts adolescents’ schooling, with few girls returning to school after childbirth. Upon deciding to carry a pregnancy to term, adolescents used several coping strategies such as relocating from usual residence, hiding until delivery and planning to put up the child for adoption upon delivery.

**Conclusions:**

Early interventions to provide adolescents with comprehensive pregnancy prevention information and to address sexual violence and poverty can prevent unintended pregnancy in adolescents. Efforts to support adolescents to positively cope with unintended pregnancy and facilitate re-entry to school are also warranted.

## Introduction

Preventing early and unintended pregnancy among adolescents is essential for their sexual and reproductive health (SRH) and successful transition to adulthood. However, nearly half (49%) of the 21 million pregnancies among girls aged 15–19 years in low- and middle-income countries are unintended [1, 2]. In Africa, about 45% of all pregnancies among adolescents are unintended in 2016, with 18% resulting in childbirth, 21% in abortions (mostly induced), and 6% in miscarriages [2]. Nearly a quarter of the unsafe abortion cases in sub-Saharan Africa (SSA) occur among adolescent girls [3]. In Kenya, the 2014 Kenya Demographic and Health Survey (DHS) showed that 47% of births among adolescents were either mistimed or unwanted [4]. The country’s adolescent abortion rate of 38 abortions per 1000 girls aged 15–19 years is one of the highest globally [5].

Previous research has documented the severe adverse health, education, social and economic consequences of early and unintended pregnancies among adolescents [2, 6, 7]. A study in six SSA countries showed that the majority of girls who reported ever being pregnant were out of school [8]. Low educational attainment among adolescent mothers contributes to a cycle of poverty, as they are unable to fully participate in the labor force or acquire greater social capital to be fully involved in their respective communities [6]. Studies also show that pregnancy-related conditions are a major cause of mortality among adolescent girls in low- and middle-income countries [9, 10]. Apart from challenges to the adolescent, early childbearing also jeopardizes the health of their infants as prior studies have shown clear evidence of higher perinatal deaths and low birth weight among babies born to mothers younger than 20 years than among those born to mothers aged 20–29 years [10, 11].

A growing body of research indicates that early and unintended pregnancy among adolescent girls is influenced by a myriad of contextual factors at the individual, community and societal levels. Several studies from high-income countries indicate that adolescent girls from families of low socio-economic status and living in poor neighborhoods are at greater risk of early and unintended pregnancies largely due to poverty and lower expectations of future economic success [12–15]. In the United States, a study by Kearney and Levine [12] showed that the combination of being poor and living in an area of greater inequality exposes young women to elevated risks of early, non-marital childbearing, potentially because of their lower expectations of future economic success. Studies that investigated the link between adolescent pregnancy and area deprivation also found that the areas with higher levels of deprivation were found to have higher adolescent conception rates [14, 16].

In Kenya, a considerable proportion of urban residents live in poor informal settlements often referred to as slums. Young people constitute about a third of the population of slums in Nairobi [17]. These young people face unique challenges as they transition to adulthood under conditions of extreme poverty; limited access to quality schools, essential services, and amenities; as well as high levels of sexual and gender-based violence [18, 19]. Research shows that people living in slums are at greater risk for early childbearing, unprotected sex, and other adverse SRH outcomes compared with their non-slums counterparts [20]. Adolescent women living in poor slum settlements are also at higher risk for both unintended pregnancy and premarital childbearing than their non-slum counterparts. An estimated 49% of pregnancies among 15-19-year-olds in Nairobi’s slums are unintended [17]. Girls reporting unintended pregnancies are more likely to be out of school, married and have lower educational attainment [19].

Health and other social risks associated with early sexual activity and pregnancy raise urgent need for interventions and programs to enhance adolescent SRH and support pregnant adolescents and adolescent mothers. It is therefore imperative to have a nuanced understanding of how adolescents deal with unintended pregnancy. However, adolescents’ experiences with, and strategies and practices for managing unintended pregnancy are under-researched. Using data from the United States, Kearney and Levine [12] investigated how adolescent pregnancies occurring outside marriage were resolved. They found that an increasing proportion of unmarried pregnant adolescents gave birth outside of marriage and/or sought abortions but fewer pregnancies led to what they called ‘shotgun marriages’, marriages that happen following pre-marital pregnancy among pregnant unmarried women [15, 21]. In their analysis of how variations in socio-economic status and income inequality contribute to variations in how adolescent pregnancies are resolved, Kearney and Levine [12] observed that adolescent girls of lower socioeconomic status in regions of high income inequality, were far more likely to "keep their baby" while adolescents of higher socioeconomic status and in regions with less income inequality had lower birth rates, the latter practicing more induced abortions.

Little is known about how adolescents in low- and middle-income country contexts resolve unintended pregnancies. Existing studies on the management of unintended pregnancy have not specifically addressed adolescents as a group [22]. Yet, for adolescents in these settings, barriers to accessing sexual and reproductive health services and structural exclusion increase their vulnerability to unintended pregnancy. Further, male partners are often excluded from studies on unintended pregnancy. The male perspective is therefore missing in the discussion around ‘intendedness’ and their influence on the pregnancy outcome [23]. Tsui et al. note that partner’s attitudes and behavior play a significant role in decision to use contraception as well as the decision to keep or terminate a pregnancy. This study addressed these evidence gaps by examining the perspectives of both male and female adolescents living in resource poor settings in Nairobi on managing unintended pregnancy. We aimed to answer two questions: 1) what are the unintended pregnancy-related experiences of adolescents in urban poor communities in Nairobi, Kenya? and 2) How are the consequences of unintended pregnancy among adolescents managed in the informal settlements?

## Methods

### Study design

This paper draws on data collected as part of a formative study—for which a report has been published—[25] that used a descriptive research design and a qualitative, case study approach.

### Study setting

This study was conducted in Korogocho and Viwandani, two slum settlements in Nairobi, Kenya, where APHRC has been running and managing the Nairobi Urban Health and Demographic Surveillance System (NUHDSS) since 2002. The NUHDSS covers 14 villages and has a population of about 65,000 individuals in the two settlements [24]. Together, the two slum settlements cover an area of about 0.97 km^2^ and are located about 7 km from each other [24]. Residents in both slums are faced with a myriad of challenges that are triggered by pervasive poverty. Like other slums [17], Korogocho and Viwandani are characterized by limited and sub-standard infrastructure, poor sanitation facilities, high levels of unemployment and high unmet service provision needs. Adolescents living in both slums are likely to be school-going adolescents (56%), not yet married (92%) and be involved in extracurricular activities such as sports clubs, boys/girls clubs or health clubs (72%) [25]. However, gender disparities in outcomes exist, with young women more likely than their male counterparts to be married (2% vs. 14%), to have dropped out of school (39% vs. 49%) or to already have a child (2% vs. 16%) [25].

### Sampling procedure

This study involved 105 male and female adolescents aged 15–19 years who participated in in-depth interviews (IDIs) and focus group discussions (FGDs). Study participants were purposively selected from households in the NUHDSS. They were diverse in terms of age, level of education, marital status and pregnancy history. We conducted 49 IDIs and eight FGDs—four in each site—with seven to eight participants per group, for a total of 56 participants. The FGDs were further disaggregated by age and sex: four with 15–17 years and 18–19 years adolescents and four each with girls and boys respectively. Thirty of the adolescents who participated in the IDIs had some secondary education; roughly half of them were not in school at the time of the study. The majority of IDI participants were not married. Thirty-five out of the 56 FGD participants were in school at the time of the study, 32 had some secondary education and 53 were not married. The socio-demographic characteristics of the participants in the study are presented in Table 1.

**Table 1:** Characteristics of in-depth interview and focus group discussion respondents.

	In-depth Interviews			Focus Group Discussions		
	Female	Male	Total	Female	Male	Total
	n = 27	n = 22	N = 49	n = 26	n = 30	N = 56
**Age**						
15	3	1	4	6	4	10
16	5	5	10	5	9	14
17	5	4	9	6	3	9
18	6	5	11	4	6	9
19	8	7	15	5	8	13
**Schooling status**						
In school	9	15	24	15	6	21
Out of school	18	7	25	11	24	35
**Education level**						
Primary	10	9	19	9	15	24
Secondary	17	13	30	17	15	32
**Marital status**						
Married	5	0	5	1	2	3
Never married	22	22	44	25	28	53
**Residence**						
Korogocho	16	10	26	15	16	31
Viwandani	11	12	23	11	14	25

Source: Mumah et al, 2014 [25]

### Data collection and management

The study was conducted in January and February 2013. A multi-national team of researchers with extensive research experience in adolescent health as well as SRH in resource-poor urban settings designed the semi-structured study guides. Interview questions primarily sought information on adolescents’ personal experiences with, as well general knowledge about, sexuality, unintended pregnancies and abortion. The interview guides were developed in English and translated into Swahili by bilingual researchers. The research team and field interviewers reviewed the English and Swahili guides to ensure consistency in meaning. The guides were pilot-tested prior to fieldwork among adolescents of similar characteristics in a slum outside of the study area.

The research team worked with field workers to recruit the participants. The field workers visited the households and introduced the study to the selected adolescents and their respective parents/guardians. Six trained and experienced male and female fieldworkers conducted the interviews in Swahili. In addition to field notes taken during the interviews and discussions, interviews and discussions were audio-recorded with the consent of the participants. On average, the group discussions lasted about an hour while the individual interviews took 35 minutes. The audio recordings were transcribed verbatim into English from Swahili by a bilingual transcriber. Two experienced research team members coded the transcripts independently using a jointly-developed codebook on NVivo software. Subsequent interpretations were compared and consensus derived on divergent opinions. The coded data were analyzed using a thematic framework approach. Key themes regarding causes and consequences of unintended pregnancy, contraceptive use, coping with unintended pregnancy, and abortion within the context of young people’s lives in the slums were generated deductively from the broad topics of the interview guides. Emerging themes from the data were also included in the framework. Word-for-word quotations are presented to illustrate key issues and themes.

### Ethical considerations

All participants above 18 years of age gave written consent to participate in the study and have the interviews audio-recorded. Interviewers also obtained parental/guardian consent for participants aged 15–17 years, who also assented to participate. Interviews were conducted in places offering auditory privacy to protect the confidentiality of responses and enhance the comfort of respondents. Population Council’s Institutional Review Board and the Kenya Medical Research Institute’s Ethics Review Committee (KEMRI) granted ethical approval to conduct the study.

## Results

Adolescents’ narratives included both personal experiences of unintended pregnancies as well as accounts of the experiences of other young people known to them. The narratives suggested that most adolescent girls’ first pregnancies were unintended, often occurring when the girls were very young and unprepared for motherhood. We summarize adolescents’ narratives around unintended pregnancy under three broad themes: causes of unintended pregnancy, consequences of unintended pregnancy and dealing with unintended pregnancy.

### Perspectives on causes of unintended pregnancy

Five sub-themes emerged with respect to the causes of unintended pregnancies: poverty, sexual violence, challenges in access and use of contraceptives, family context, and peer pressure. Four of the major themes are described in detail below.

#### Poverty

In both the IDIs and FGDs, many of the adolescent boys and girls cited household poverty as an important driver of early sexual activity and, subsequently unintended pregnancy among girls. Adolescents noted that many girls in the slum engaged in sexual relationships with boys or older men in return for money to pay school fees and buy, among other items, food, clothing, and toiletries. As illustrated in the following excerpt from a participant in an FGD with 15- to 17-year-old girls in Viwandani, such transactional relationships placed girls in difficult situations where they cannot negotiate for condom use or contraceptives, increasing their vulnerability to unintended pregnancies:

*There is poverty*. *Some girls are forced to look for boys and older men*. *Maybe he has refused to use the condom or you also don’t know about family planning methods which is also not good for you*. *And one might end up getting pregnan*t

#### Sexual violence

Adolescents’ accounts also suggested that sexual violence was a common reason why unintended pregnancies occurred. For some, sexual violence was associated with alcohol and drug use and often meant that sexual intercourse was unprotected. As a 19-year-old girl from Viwandani explained:

*Some might happen by accident*, *for instance*, *someone has smoked bhang* [marijuana] *and is unaware of his action; he might take you by force … He might rape you and you may not know whether you are pregnant or no*t

#### Challenges in access and use of contraceptives

Data from IDIs and FGDs indicated that sexual encounters among adolescents were mostly unprotected despite some knowledge about contraception. Reportedly, contraceptives were inaccessible and unaffordable to adolescents. Inadequate knowledge of contraceptives, and shame or stigma experienced by adolescents who sought to obtain contraceptives also were identified as significant barriers to the use of contraception among adolescents. A 16-old-year old boy in Korogocho noted:

*It is not easy for small [young] people like us to go to a pharmacy to ask for condoms or pills*…

Respondents often talked of limited choices of methods for adolescents and problems in accessing the services. Among adolescents with personal experience with contraception, many mentioned pills, condoms and injectables. Mostly, adolescents reported that the choice of contraception was not based on expert opinions from service providers but rather on what their peers were using. Reports of contraceptive failure among adolescents were also common. Respondents noted that some adolescents used contraceptives inaccurately and inconsistently, which often resulted in unintended pregnancies.

Moreover, adolescent responses showed their distrust as well as dissatisfaction about contraception. Dissatisfaction stemmed partly from personal experience as well as experiences of friends and peers. Misconceptions about contraceptive use and perceived side effects therefore had a negative impact on young peoples’ willingness to use contraception. As illustrated from the following quote from an 18-year-old girl from Korogocho, misconceived side effects included the fear of “growths” [also cited in reference 27].

*…So condom also is risky if you use for a long time you can be infected with a disease*, *growth in the stomach*, *so we don’t know which protection we will use*? *We don’t know whether we just go for prayers*?

#### Family context

Parent-child relationships encompassing parental support, control or supervision for adolescents might be protective against early and unintended pregnancies [27–29]. Adolescents noted that lack of parental support and guidance, as well as exposure to household conflicts increase girls’ risk of early sexual debut and risky sexual behavior. According to participants, some girls ran away from home after conflicts with their parents, usually to live with male companions. Some adolescents were also reportedly driven to have sex out of curiosity or defiance stemming from parents’ disapproval of relations with members of the opposite sex. Young people felt that they did not get guidance on sexuality from their parents, especially on menstruation, maturation and consequences of sex and were left to learn about sex on their own. Data suggested that limited parental guidance could partly be attributed to parents’ daily busy routines that leaves little time for them to interact with their children.

In the FGDs, participants acknowledged that some parents do not question where their adolescent girls get money, implying that the adolescents would continue having transactional sexual relationships, with some of the parents even benefitting from them. Narratives suggest that for some teenage girls, engaging in sex-for-money relationships to support household expenses were encouraged by parents. As noted by a boy in a FGD with boys aged 15–17 years in Korogocho;

*Some parents also just receive money from their daughters*, *not knowing how the daughters got the money; because of poverty when their daughters bring them like two hundred shillings the mother will receive it and rush to buy cabbage*, *instead of asking her how she got the money (FGD Boys*, *Korogocho*, *15–17 years*).

Congested housing and living arrangements also expose adolescents to sexual activities by parents or guardians. Some adolescents share single rooms with their parents or guardians and witness sexual encounters among the adults. As a result, many young people learn about sex and/or are influenced to engage in sexual activities early on. An 18-year-old girl from Viwandani painted a scenario where adolescent might live with single parents in a single room.

*…they are single mothers like my mother and she brings her boyfriend at home and it is one room… So you tend to know these things early in life*. *When your mother brings a man in the room you know… you hear things like…these things happen so you feel in other words you are exposed to these things very early in life*. *Maybe you heard your mother having sex and then…So when you sit there you get those feelings*.

### Perspectives on consequences of unintended pregnancy

#### Dropping out of school

Participants noted that dropping out of school was common in the slum communities, particularly when parents or guardians could no longer afford to pay for their schooling, due to pregnancy and due to drug abuse or behavior related problems for boys. Narratives indicated that unmarried young girls who became pregnant dropped out of school either due to shame and stigma or would sometimes be expelled from schools. An 18-year-old boy in an IDI in Korogocho noted:

*They can’t go to school… in our school they are chased*. *We are told*, *‘no coming to school two-in-one*’.

In the FGDs with adolescent girls, participants narrated experiences of their friends who dropped out of school due to pregnancy and could not go back even after childbirth. Some described getting pregnant while still in school because they were “cheated” by boys and men (and even teachers) who have money. Others narrated the difficulty of going back to school after childbirth or the difficulty girls face in concentrating on their education once they have given birth. A participant in an FGD with girls aged 15–19 years from Viwandani stated:

*You know when you get a baby and you are told to go back to class you will spend your time thinking about your baby*.

A common opinion among participants was that pregnancy-related school drop-out affects mostly girls while boys are rarely affected. Among boys, dropping out of school was driven by a sense of responsibility to take care of the new family. Adolescents, however, mentioned that some pregnant girls and boys who impregnated girls dropped out of school due to shame and stigma. Both boys and girls were regularly taunted and stigmatized by their peers. For some adolescents, dropping out of school was due to parents discontinuing support as a consequence of the girls getting pregnant or the boys impregnating girls. There were also instances where boys who impregnated a girl reportedly drop out of school to get married and take care of the family.

#### Loss of family and community support

Parents’ and families’ reactions to a girl’s pregnancy varied. Some families isolated and limited association with the girl, even within the household, beating them as punishment and withdrawing support for their education. A 17-year-old girl from Korogocho explained that dropping out of school was more common for girls in the slums.

*You know life in the ghetto*, *unlike these other people who are rich maybe one may become pregnant and is allowed to give birth and then go back to school*. *Here*, *the moment you are pregnant*, *the way your parents struggle*, *they will feel like you have really insulted them*, *it is like they were wasting time on you; you will give birth and there is no going back to schoo*l

Adolescents reported that reactions from the community towards a pregnant teen tended to be negative and judgmental. Pregnant girls were shunned, disparaged, forbidden to talk to other girls because they were considered to be ‘girls of loose morals.’ Some girls even reported losing some of their close friends due to the pregnancy. A 17-year-old girl from Korogocho who was pregnant while in school narrated her experience:

*They say that you are a prostitute*, *you like men…*. *My best friend with whom I was with at school at that time didn’t talk to m*e

### Dealing with unintended pregnancy

Adolescent girls described the experience of finding out they are pregnant using words such as ‘shocking’, ‘disbelief’, and ‘dismay.’ They noted that immediately after finding out their pregnancy, the dread of how to resolve the pregnancy takes over. They pointed out that a myriad of factors influence a girl’s decision-making in dealing with unintended pregnancy. Notably, her relationship with her boyfriend or the putative father as well as the girl’s thoughts about how her parents would react to the news of her pregnancy greatly influence her decisions. To many girls, breaking the news of an unintended pregnancy to male partners was a first major challenge. Adolescents noted that there were instances where some girls were physically assaulted when they announced the pregnancy to the male partners. Some girls therefore devised ways of communicating indirectly to their partners such as by hinting about signs of pregnancy during conversations. A 17-year-old boy explained:

*And she will not come directly to him and say that she is pregnant but she will be like yesterday I vomited so much…You tell her it could be flu…The next day it is loss of appetite and so she will keep giving you the signs and if you are clever you will know that she is pregnant*…

Ultimately, the decision to carry a pregnancy to term or to have an abortion was influenced by a range of factors. These factors are described in greater detail below.

#### Induced abortion

Responses from IDI participants suggest that abortion was common in the community, and the decision to have an abortion was often driven by fear of parents’ reaction to the pregnancy, fear of being stigmatized as an unmarried pregnant girl, disruption to their social life, desire to continue school and the perceived cost of raising a child. Participants in the FGDs agreed that abortion was the ultimate choice when one’s boyfriend denies responsibility and when the perceived reaction of parents is negative. As noted by a participant in an FGD with girls aged 15–17 years in Viwandani:

*because may be you don’t have the ability at home*, *the boy has deserted you*, *or you don’t want your parents to know because they are so harsh*, *you feel the best way out is abortio*n

Some participants reported that there are instances where parents play an important role in the decision to terminate a pregnancy. For example, when parents find out about the pregnancy, there are those who support abortion to avoid embarrassment to the family. A 19-old-male in Korogocho noted:

*There is no way they will get into problems with their parents*. *In fact*, *the parents are the ones who will take care of the expenses because they often insist that they don’t want the pregnancy and if you want to keep it*, *you will have to move out of their home*.

However, participants noted that the opportunities to have open discussions about abortion with parents are often limited. As such, they reported that most girls seek advice about abortion from their close friends, particularly those who had an abortion themselves.

In both the IDIs and FGDs, adolescents emphasized that fear of disruption to the girls’ education is a major reason for abortion among school girls. Moreover, adolescents maintained that most abortions were either self-induced or procured with assistance from herbalists, and unskilled providers. Data from the IDIs suggested that many methods are used by adolescents to procure abortions. The decision on which methods to use to terminate a pregnancy was influenced by several factors including cost, confidentiality, convenience, and peer influence. Efficiency and safety were the main considerations when choosing the method of abortion but some adolescents tended to choose those perceived to be quick and readily available. Adolescents considering an abortion would often confide in close friends particularly those with prior abortion experience. As illustrated in the following quote from a 15-year old girl in Viwandani, the decision to procure an abortion or not was not always straight forward:

*Maybe she is undecided whether to have an abortion or not*. *She comes to me (as a friend) and I tell her it is not good*. *Another friend tells her to go ahead and terminate i*t

#### Carrying the pregnancy to term

For some adolescents, the difficulties with accessing safe abortion due to the legal limitations and availability of pregnancy termination services, fear of the dangers of, and lack of money to pay for an abortion may tilt the decision in favor of carrying the pregnancy to term. Narratives showed that abortion decision making is challenging due to the health risks and moral dilemma it carries. In both the IDIs and FGDs, respondents narrated how stories of girls becoming seriously ill or dying from abortion complications cause fear. The fear that abortion causes infertility is also widespread and dissuades girls from seeking abortion. An 18-year-old girl from Viwandani said:

*So you hear that if you abort you will never get any other child in your life and you will live like that without a child*.

Even if an abortion is to resolve an unintended pregnancy, the fear that someone may die in the process is common due to the clandestine nature of abortion services in the slums, thus some decide to carry the pregnancy to term. A 19-year-old mother from Korogocho, recounted her experience seeking to have an abortion in an FGD. She said:

*Like I went to do abortion*, *and when I reached there and saw how the tools were arranged*, *I ran back home*. *Now*, *when I watch my son walking I just ponder and say ‘what if I had aborted my child’*? *So I am now happy to be called a mothe*r

Others pointed out that even though services are clandestinely provided, the cost of having an abortion is unaffordable for girls, and this continues to rise with the gestational age. This was highlighted by a 19-year-old girl from Viwandani, who said:

*You find out from someone who has done it before*, *how much is it to have an abortion*? *She will tell you either Sh5*, *000 [USD 50] or maybe Sh10*, *000 [USD 100] and you are like where will I get all this money*? *Let me just stay like that and keep the pregnancy*.

The decision to keep the pregnancy also largely depended on the availability of support from the boyfriend, parents, close friends and others. Many adolescent girls would decide to keep the pregnancy if there was parental resistance to abortion, parental willingness to provide financial support for the child, or assurances of marriage or financial support for the child from the father or another male partner. For some young girls, the decision to keep the pregnancy was also influenced by availability of external assistance, such as maternal health care vouchers that could allow them afford hospital visits, delivery, and or any complications with the pregnancy they may face.

Adolescent girls who decided to carry their pregnancies to term were noted to face several challenges. The challenges often revolve around meeting the child’s basic needs, avoiding societal stigma, parental anger and abuse, being disowned and thrown out of their home by parents. To cope with these challenges, girls reportedly deploy various strategies including moving away from their usual places of residence, planning to give the child up for adoption when delivered, and hiding the pregnancy until childbirth.

In a few instances where unintended pregnancies were carried to term, some girls were noted to eventually give up the child for foster care, or some extreme cases, abandon or even kill the child. In talking about foster care, a girl aged 18 years from Korogocho said:

*let’s say you are pregnant and you didn’t abort the baby*, *but you gave birth*…*and since you want to go back to school*…*We have a charity home here… you can take the baby there and then you can go back to school*…

Reaction to an unintended pregnancy was disbelief, shame, embarrassment, resignation, anger and regret. As such, when girls decide to carry pregnancy to term, some would resort to hiding till the baby was born due to shame and embarrassment. Some girls opted to stay indoors and refrain from community activities to avoid the embarrassment and hostility. An 18-year-old from Korogocho, who had her first child at 17 years explained:

*…she feels ashamed and doesn’t want to come out*, *for example*…*So*, *maybe she wants to stay with the pregnancy until nine months are over and then she will…She will start to come outside once she gives birth*.

Adolescents explained that pregnant girls adapt to the changed relations in the family in different ways: Some girls became more cooperative with their parents to gain their support during the pregnancy. Other girls moved from the city homes to their rural homes and lived with relatives or friends until childbirth. Most of the girls who did not get any form of support in their homes or from their partners did casual jobs such as house helps, in salons or selling items to sustain themselves and their child.

#### Boys’ responses to unintended pregnancy

Boys’ responses to a pregnancy included pride, outright denial, ambivalence, threat of violence, embarrassment, resignation and acceptance. In situations where boys accepted and took responsibility for the pregnancy, narratives linked this to the pride these boys felt that they made a girl pregnant. Data showed that this pride comes from the recognition they receive from their peers about their new status of fatherhood, which positively confirm their manhood and potency. A 19-year-old boy from Korogocho stated:

*Maybe he wants to get her pregnant so that he can be responsible or for people to know that he is an adult and can fertilize; you may hear your friends say that yesterday he got someone pregnant… he tells you that your sperms cannot do anything*.

This sense of pride seemed to be heightened if the impregnated girl was considered a ‘difficult catch’. In accepting the pregnancy, boys found different ways to raise money to support the pregnant girl. Responses showed some boys resorted to crime, sought employment, or left the child with his parents. Further, though some of the young boys reported marriage as a strategy, data showed that marriage was not always a voluntary decision for the boy, rather, some boys were forced (usually by the girl’s parents) to take that step.

In some cases, denial stemmed from suspicion that girls had multiple sexual partners and could not be trusted. Some boys reportedly deny pregnancies because they used condoms, while others claim that they started the relationship while the girl was already pregnant. This distrust is illustrated in the following quote from a boy in an FGD with boys aged 17–19 years in Korogocho.

*Somebody cannot say that she is pregnant for you*, *how will you know whom she has met with before you*. *You know here these young girls will just ‘open up for you’ when she knows that she has slept with an adult*. *So that you become the one responsible for her pregnancy [laughter in the background*]

In some instances, waiting for the birth of the baby, to ascertain their paternity is how uncertainty was dealt with. Paternity, however, is ascertained usually by checking who the baby ‘looked’ like. A 19-year-old boy from Korogocho said:

*“Some of them will only accept after you have given birth to a child who resembles them*, *and that is when they will accept the child is really their*s

Narratives suggested aggression from boys towards girls and insistence on an abortion. In some instances, however, boys who wanted the girl to keep the pregnancy would invoke religious beliefs as a reason against abortion. As illustrated in the follow quote from a participant in an FGD with 17- to 19-year-old boys, for some boys, abortion was not an option because they could financially support the girl and the child:

*Now that I am working*, *I know I will not miss my eight thousand shillings and so I will come and have a talk with her*. *Tell her the position and ask her not to terminate the pregnancy*. *You and I can take care of that pregnancy and the girl will also feel there is no point in having an abortio*n

Narratives suggested that boys who impregnate girls worry a lot about the cost of raising up a baby, especially because the majority are usually still dependent on their parents for their own basic needs, including schooling. Many boys consider themselves too young for fatherhood. This is particularly worrisome in instances where the girl’s family pressurizes the boy to take responsibility of raising the baby.

## Discussion

The study analyzed qualitative data collected from young people living in Nairobi’s urban slums. Young people growing up in Kenyan slums face a myriad of challenges and are at high risk of unintended pregnancy. Findings reveal that unintended pregnancies among young people in the slums are common and mainly attributed to poverty, sexual violence, and poor access to SRH services.

We found that the need to access basic needs may push girls to engage in unprotected transactional sex. As adolescents in our study explained, many girls in the slum engage in sexual intercourse with boys or older men in return for money to buy food, pay school fees, and to purchase other material needs such as clothing and toiletries where parents or guardians are not able to provide. Similar findings have been reported in other low-income settings in SSA [26]. Transactional sex is noted to be a major driver of early and unintended pregnancy among adolescent girls in the region [27–29]. According to some scholars [30], transactional sex takes away girls’ power to negotiate contraceptive use. Moreover, transactional sex with multiple partners can leave adolescent girls unsure of the men responsible for their pregnancy and provides men the excuse to reject paternity, thereby increasing the challenges a girl would face if she becomes pregnant [27, 28, 30].

Adolescents’ accounts suggest that many of them receive information about contraception from peers rather than health providers. Limited access to accurate information about contraception may explain the widespread myths about perceived side effects and health concerns of contraception. These myths and misconceptions act as barriers to contraceptive use. This finding resonates with the work of Gueye et al and Ochako et al [31, 32] who found that peers and young people’s social networks played a critical role in transmitting myths and misconceptions about contraception. Addressing adolescents’ concerns about contraceptives is important as significant proportions of young users discontinue contraceptive use shortly after adoption due to method-related dissatisfaction [33].

Evidence shows that about 79% of unintended pregnancies occur because effective contraception was not being used [34]. Similarly, social and cultural contexts play important roles in the choices that individuals make, be it the timing and type of relationships formed, how partners negotiate their interests within relationships, acceptability of gender-based violence or even use of contraception [23].

It is possible that the lack of deliberate pregnancy planning is due to the irregular and unstable nature of the sexual relationships among young people. It could also be indicative of poor access to accurate information and services to cater the SRH needs of young people as Lince-Deroche et al (2015) found in South Africa [35]. In Kenya, the 2014 DHS showed that 22% of girls 15–19 have an unmet need for contraception for spacing purposes. As a result, almost half of the pregnancies among adolescents in Kenya and in the slums are unintended [17,36]. Enhancing existing service provision channels to provide accurate information and services on a wide range of methods to capture the diverse needs of these group is therefore needed.

This study highlights school disruption as a major consequence of an unintended pregnancy, with few opportunities available to girls [or boys] to reenter school after childbirth. Though the Kenyan government has a school re-entry policy to facilitate return to school after childbirth, there is minimal evidence that this is being practiced, with re-admission of pregnant learners still dependent on school administrators [37,38]. It is estimated that 13,000 girls drop out of school annually in Kenya due to unintended pregnancy [39]. Further, national data show the majority of ever pregnant girls are out of school in Kenya [8]. Even among our study community, 69% of young women who had ever had a child were out of school [19]. The links between early and unintended pregnancy and school drop-out is indicative of a need for the education sector to be more responsive in meeting the needs of pregnant learners, and putting the school reentry policy of the Kenyan government into actual practice.

Similar to a previous study on the management of unwanted pregnancy among women in Nairobi, Kenya [22], we found that pregnancy management was multi-layered, with adolescents experiencing a range of emotions and employing diverse coping strategies. Overall, adolescents with unintended pregnancy grappled with two options: carrying it to term or procuring an abortion. We found that adolescents in urban slums favored induced abortion as a strategy for dealing with an unintended pregnancy. However, the decision to procure an abortion was not always straightforward and was influenced by different factors including embarrassment, stigma or shame and school disruption. Findings suggest that most abortions are carried out by unskilled providers increasing the risk for negative health outcomes. The complexity of decision making may also lead to later term abortion, which may pose greater health risks. These results may explain national statistics showing that adolescents present for post-abortion care for later-term pregnancies [40] and are more likely to present with moderate and severe complications [41]. Taken together, these findings suggest the need for programs to support adolescents to positively cope with unintended pregnancies.

This study has some limitations. First, respondents recruited for the study might have not experienced unintended pregnancy although the study assessed their perception, experiences with unintended pregnancy and its copying strategies. Second, the study is limited to two urban informal settlements. Adolescents’ experiences in these settlements may differ markedly from those of adolescents in other settlements. Notwithstanding, the findings reported in this paper offer insights into the concerns that adolescents grapple with when they experience unintended pregnancy particularly in contexts marked by pervasive poverty.

## Conclusion

Adolescents in urban slums face significant challenges that increase the risk of early and unintended pregnancy and, consequently, unsafe abortion. These challenges include poverty, limited access to accurate and comprehensive information about contraception, limited access to SRH services, high vulnerability to sexual violence, and limited parental support. Addressing these structural issues using a multi-sectoral approach that combines initiatives to increase access to education, alleviate poverty, improve parent-child interactions, and enhance access to health information and services may have long-term impacts on reducing the vulnerabilities to negative outcomes as young people navigate their transition into adulthood in a high-risk setting. Programs to support pregnant adolescent and adolescent mothers are also needed.

## Supporting information

S1 Checklist (PDF)

## List of acronyms and abbreviations

APHRC: African Population and Health Research Center
DHS: Demographic and Health Survey
FGDs: Focus Group Discussions
IDIs: In-depth Interviews
NUHDSS: Nairobi Urban Demographic and Health Surveillance System
SRH: Sexual and Reproductive Health
SSA: sub-Saharan Africa
WHO: World Health Organization
